# Conservation and distribution of the DRACH motif for potential m^6^A sites in avian leukosis virus subgroup J

**DOI:** 10.3389/fvets.2024.1374430

**Published:** 2024-04-12

**Authors:** Jun Ji, Xinhao Mu, Shuqi Xu, Xin Xu, Zhibin Zhang, Lunguang Yao, Qingmei Xie, Yingzuo Bi

**Affiliations:** ^1^Henan Provincial Engineering Laboratory of Insects Bio-reactor, Henan Provincial Engineering and Technology Center of Health Products for Livestock and Poultry, Henan Provincial Engineering and Technology Center of Animal Disease Diagnosis and Integrated Control, Nanyang Normal University, Nanyang, China; ^2^College of Animal Science, South China Agricultural University, Guangzhou, China

**Keywords:** avian leukosis virus subgroup J, m^6^A, DRACH motif, RNA secondary structure, comparative analysis

## Abstract

*N*^6^-methyladenosine (m^6^A) methylation is an internal post-transcriptional modification that has been linked to viral multiplication and pathogenicity. To elucidate the conservation patterns of potential 5′-DRACH-3′ motifs in avian leukosis virus subgroup J (ALV-J), 149 ALV-J strains (139 isolates from China; ALV-J prototype HPRS-103 from the UK; and 9 strains from the USA, Russia, India, and Pakistan) available in GenBank before December 2023 were retrieved. According to the prediction results of the SRAMP web-server, these ALV-J genomes contained potential DRACH motifs, with the total number ranging from 43 to 64, which were not determined based on the isolation region and time. Conservative analysis suggested that 37 motifs exhibited a conservation of >80%, including 17 motifs with a grading above “high confidence.” Although these motifs were distributed in the U5 region of LTRs and major coding regions, they were enriched in the coding regions of *p27*, *p68*, *p32*, and *gp85*. The most common m^6^A-motif sequence of the DRACH motif in the ALV-J genome was GGACU. The RNA secondary structure of each conserved motif predicted by SRAMP and RNAstructure web-server was mainly of two types—A–U pair (21/37) and hairpin loop (16/37)—based on the core adenosine. Considering the systematic comparative analysis performed in this study, future thorough biochemical research is warranted to determine the role of m^6^A modification during the replication and infection of ALV-J. These conservation and distribution analysis of the DRACH motif for potential m^6^A sites in ALV-J would provide a foundation for the future intervention of ALV-J infection and m^6^A modification.

## 1 Introduction

As a representative member of the 11 subgroups of avian leukosis virus (ALV-A–K) that have been recognized to date, ALV subgroup J (ALV-J) is an oncogenic virus that is associated with significant morbidity and causes avian leukosis, thus affecting the poultry industry worldwide (1). The genome of ALV comprises a single positive-strand linear RNA dimer, and its provirus presents a gene structure characteristic of C-type retroviruses, spanning a total length of 7–8 kilobases (2). The genes encoding the group-specific antigen (*gag*), polymerase (*pol*), and envelope glycoprotein (*env*) are located within the central coding region of the viral genome molecules (3). The noncoding region, mainly contained the long terminal repeat (LTR), is a pair of identical sequences of DNA situated at both ends of the genome, which comprises the 3′ unique region (U3), a short repeated region (R), and the 5′ unique region (U5) (4). These regions play crucial roles in viral replication, RNA processing, and integration of the virus into the host genomes (5, 6).

Currently, several research projects are dedicated to the elucidation of the pathogenesis of ALV-J, which is mainly related to its tumorigenic effect, immunosuppression, and decreased performance in chickens and layers (7). After the integration of the virus genome sequence into the host genome, the viral LTR plays a key role in the activation of the cellular proto-oncogenes, a process known as promoter insertion, which ultimately leads to multiorgan hemangioma and myeloid leukosis (8). Recent studies have shown that the aberrant expression of endogenous microRNAs (miRNAs), circular RNAs (circRNAs), and long noncoding RNAs (lncRNAs) caused by ALV-J infection is closely related to avian leukosis virus-induced tumorigenesis (9–12). These regulating effectors are mainly related to various activate/inactivate signaling pathways and differential expression change of genes at mRNA and protein levels.

Historically, DNA has been acknowledged as the carrier of genetic information, whereas mRNA has been identified as the central molecule that connects DNA to proteins and is crucial for the transmission of genetic information during various biological processes (13). To date, >100 chemically distinct modifications have been reported in cellular RNAs. Notably, in eukaryotic organisms, the presence of a modified 5′-cap at the 5′-end and a polyadenylate (poly(A)) tail at the 3′-end of mRNAs is crucial for the regulation of mRNA transcription (14). The prevalent alterations observed in mRNA mainly include *N*^1^-methyladenosine (m^1^A), 7-methylguanosine (m^7^G), *N*^5^-methylcytosine (m^5^C), and the prominent modification *N*^6^-methyladenosine (m^6^A) (15).

Generally, the m^6^A modification of RNAs occurs via a hetero-multimeric complex of nuclear methyltransferases (writers); in turn, this dynamic modification can be reversed by a group of demethylases (erasers), whereas a third group of proteins, known as readers, preferentially recognize the binding of m^6^A (readers) to methylated RNA and confer downstream functions (16). Therefore, aberrant m^6^A modifications and their related enzymes/proteins may be the best strategy for understanding the epigenetic mechanisms underlying the expression of important viral genes during the course of viral infection (17). Recently, there has been an increasing amount of research on the relevance of the m^6^A changes in viral replication and virus–host interactions in relation to virus induced illnesses in humans and animals (17). However, the functional characteristics of the m^6^A-mediated regulation of the RNAs/transcripts of DNA/RNA viruses remain unclear. According to the modification process of m^6^A, the core step consists in the recognition of the target genes, which calls for the accurate identification of RNA m^6^A sites (18). Numerous independent reports have revealed that the 5′-DRA*CH-3′ (where D stands for non-cytosine base; R for purine; H for non-guanine base; and A* for the methylatable adenosine) consensus motif is restricted for m^6^A binding; however, the mapping of these sites in viral RNAs/transcripts remains challenging and cumbersome (19, 20). Consequently, we aimed to use publicly accessible ALV-J genome sequences to predict m^6^A locations and study their evolutionary conservation, to stimulate research on m^6^A modification in ALV-J.

## 2 Materials and methods

### 2.1 Genome sequence collection of ALV-J

The complete genome sequences of 149 ALV-J strains submitted before December 2023 were retrieved from GenBank (Supplementary Table 1). These ALV-J strains included 139 isolates from China, the ALV-J prototype HPRS-103 from the UK, and nine strains from the USA, Russia, India, and Pakistan.

### 2.2 Prediction of the DRACH sites

The genome of ALV-J comprises a positive-strand linear RNA, and the coding regions of the three primary genes (*gag*, *pol*, and *env*) are continuous or overlapping. Moreover, previous studies have shown that m^6^A modification is primarily distributed within the coding regions, close to the start or stop codons and near the beginning of 3′-untranslated region (3′-UTR) or ending of 5′-UTR in mRNA transcripts derived from chickens that were negative/positive for ALV-J (21, 22). Therefore, the m^6^A-modification sites in the genome sequences and coding sequences of the 149 ALV-J strains were predicted via the SRAMP^1^ online server using the “Full transcript” and “Mature mRNA” modes, respectively (23). For additional validation, the RNA secondary structure for each motif predicted in this manner was also summarized.

### 2.3 Sequence alignment and conservation analysis of potential m^6^A motifs

Multiple sequence alignments of the genome and coding gene sequences of ALV-J strains were performed using MEGA version 11 (24). Based on a comparison of the prototype strains HPRS-103 and NX0101 (GenBank accession no.: DQ115805, representative Chinese ALV-J strain caused myeloma), the conservativeness and diversity of all predicted DRACH motifs were visualized using TBtools-II software (v2.028) (25). The distribution of conserved m^6^A-modification sites in ALV-J genomes was visualized via lollipop-plotting using ChiPlot^2^, and the genome structure of ALV-J was illustrated using IBS software (26).

### 2.4 RNA secondary structure analysis

According to the conservation analysis and rating scores of the predicted motifs, to further evaluate the accuracy and potential modification mode of each m^6^A motif, the RNA secondary structure of the flanking sequences of each m^6^A motif was re-predicted using the RNAstructure web-server^3^ (27).

## 3 Results

### 3.1 Prediction of DRACH motifs in ALV-J genomes

Based on the prediction results obtained for the 149 ALV-J genome sequences, the genomes of these strains contained potential DRACH motifs, with the total number ranging from 43 (GD1411-1; accession NO., KU500038) to 64 (GD16FS01; accession NO., MT538237) (Supplementary Table 1), which were not determined based on the isolation region and time. According to the rating grades obtained via SRAMP prediction, the proportion of motifs with “low confidence,” “medium confidence,” “high confidence,” and “very high confidence” grading was 23.76, 36.85, 34.53, and 4.87%, respectively.

### 3.2 Conservation pattern of the predicted DRACH motifs

**Figure 1:**
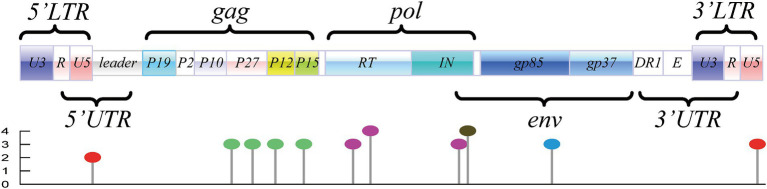
Genome structure and distribution of potential m^6^A motifs of ALV-J (represented by the ALV-J prototype strain of HPRS-103; GenBank accession number: Z46390.1). Motifs located in LTRs are marked with “red lollipop,” motifs located in the coding region of *gag* are marked with “green lollipop,” motifs located in the coding region of *pol* are marked with “purple lollipop,” motifs located in the coding region of *env* are marked with “blue lollipop,” and the motif in the overlapped coding region is marked with “black lollipop.”

Genome construction revealed that the potential m^6^A modification sites were distributed throughout the entire genome, as displayed in Supplementary Table 1 and Figure 1. In terms of conservation analysis and rating grade of the predicted DRACH motifs, 37 motifs exhibiting a conservation of >80% are summarized and listed in Table 1; these motifs comprise 17 motifs with a grading above “high confidence,” as indicated by SRAMP. Although these motifs were distributed in the U5 region of LTRs and major coding regions, they were enriched in the coding regions of p27, p68, p32, and gp85 (Table 1).

**Table 1:** List of the conserved motifs predicted in ALV-J genomes.

Position	Genome region	Sequence	Conservation	Rating grade	RNA 2nd structure
273^a^	5′-LTR (U5)	GG **A** CC	87.25%	Moderate	A–U pair
1,265	gag (p 10)	UG **A** CU	89.93%	Moderate	Hairpin loop
1,344	gag (p 27)	GG **A** CC	100.00%	Moderate	Hairpin loop
1,354	gag (p 27)	GG **A** CC	99.33%	Moderate	Hairpin loop
1,383	gag (p 27)	AG **A** CU	88.59%	High	Hairpin loop
1,402	gag (p 27)	GG **A** CC	93.29%	Moderate	A–U pair
1,506	gag (p 27)	GG **A** CC	88.59%	Low	A–U pair
1,621	gag (p 27)	GG **A** CU	96.64%	High	A–U pair
1,892	gag (p 27)	UG **A** CU	95.30%	High	hairpin loop
2,002	gag (p 27)	AG **A** CU	82.55%	Moderate	A–U pair
2,094	gag (p 12)	GG **A** CA	81.88%	Moderate	A–U pair
2,151	gag (p 12)	GG **A** CA	97.32%	High	A–U pair
2,229	gag (p 12)	GG **A** CA	92.62%	High	A–U pair
2,444	gag (p 15)	GG **A** CU	95.30%	High	A–U pair
2,456	gag (p 15)	GG **A** CA	95.30%	Moderate	A–U pair
2,861	pol (p68)	GG **A** CA	84.56%	Low	A–U pair
3,670	pol (p68)	GG **A** CC	98.66%	High	Hairpin loop
3,710	pol (p68)	GA **A** CA	97.32%	Moderate	Hairpin loop
3,734	pol (p68)	GG **A** CA	97.32%	High	A–U pair
3,740	pol (p68)	GG **A** CU	95.30%	Very high	Hairpin loop
4,236	pol (p68)	UG **A** CU	85.91%	High	Hairpin loop
4,277	pol (p68)	GG **A** CA	84.56%	Moderate	A–U pair
4,633	pol (p32)	AG **A** CU	97.99%	Moderate	A–U pair
5,003	pol (p32)	GA **A** CU	94.63%	Moderate	A–U pair
5,105	pol (p32)	GG **A** CC	95.30%	Moderate	Hairpin loop
5,130	pol (p32)	AG **A** CA	81.88%	Low	Hairpin loop
5,200	pol (p32)	GG **A** CA	96.64%	High	Hairpin loop
5,245	pol (p32)	GG **A** CA	82.55%	High	A–U pair
5,268	pol (p32)	UG **A** CU	89.93%	Low	Hairpin loop
5,311	pol/env	UG **A** CU	93.96%	High	Hairpin loop
5,342	pol/env	GG **A** CU	87.92%	Very high	A–U pair
5,866	env (gp85)	GG **A** CU	94.63%	High	Hairpin loop
5,894	env (gp85)	UG **A** CU	81.21%	Low	A–U pair
6,220	env (gp85)	GG **A** CU	92.62%	High	A–U pair
6,318	env (gp85)	GG **A** CA	90.60%	Moderate	A–U pair
6,325	env (gp85)	UG **A** CU	83.22%	Low	A–U pair
7,791	3′ LTR (U5)	GG **A** CC	84.56%	High	Hairpin loop

a The position and genome region of each motif are based on the sequence of the ALV-J prototype strain of HPRS-103; GenBank accession number: Z46390.1. The bold and underlined “A” stands for the methylatable adenosine.

**Figure 2:**
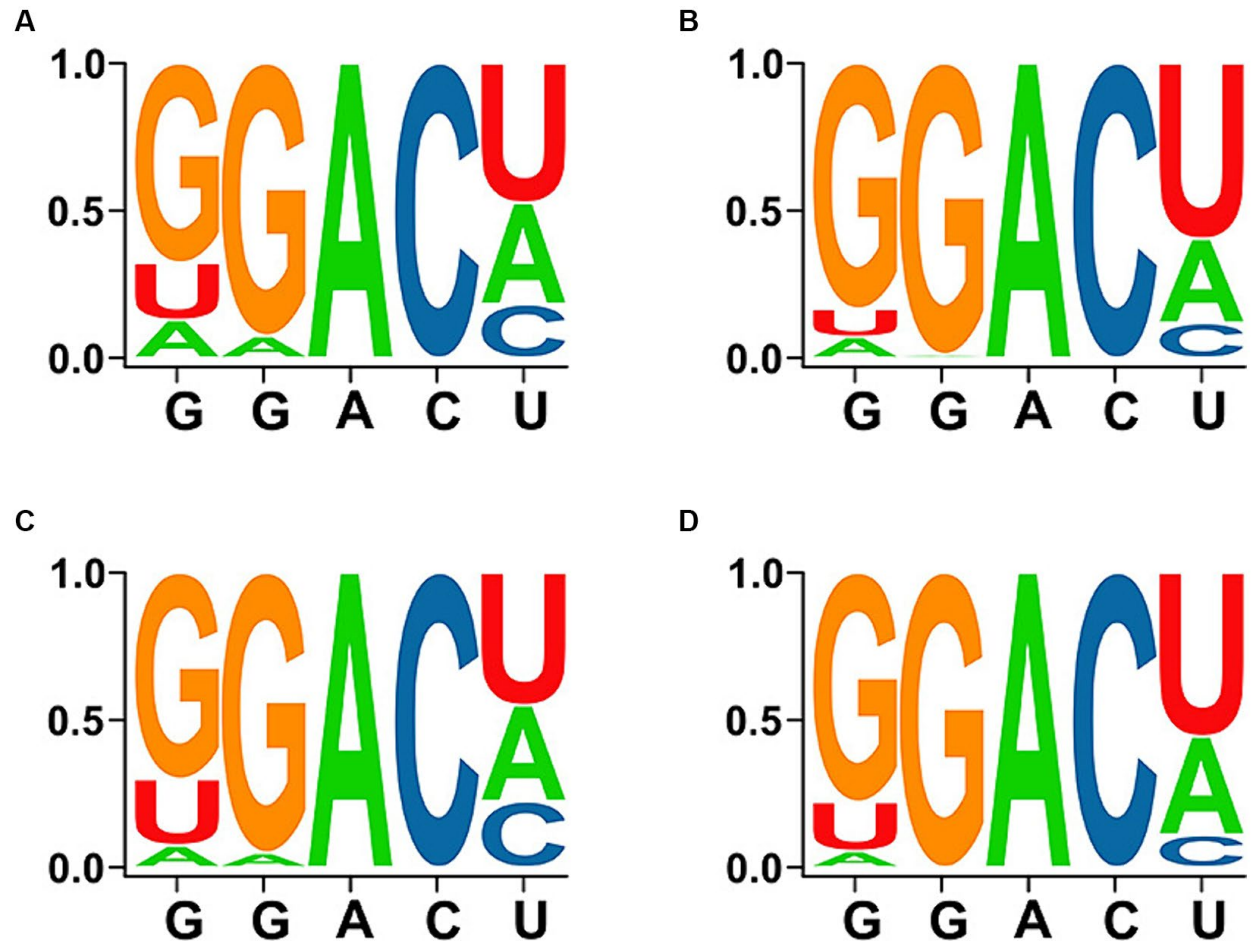
Diversity and conservation of DRACH sequences in the motifs. **(A)** Total predicted motifs. **(B)** Motifs with “high/very high confidence.” **(C)** Motifs with a conservation of >80%. **(D)** Motifs with a conservation of >80% and “high/very high confidence.”

Meanwhile, the diversity of all motifs, motifs with “high/very high confidence,” motifs with a conservation of >80%, and motifs with a conservation of >80% and grading of “high/very high confidence” are displayed in Figure 2, which revealed that GGACU was the most common m^6^A-motif sequence.

### 3.3 RNA secondary structure for conservative DRACH motifs

**Figure 3:**
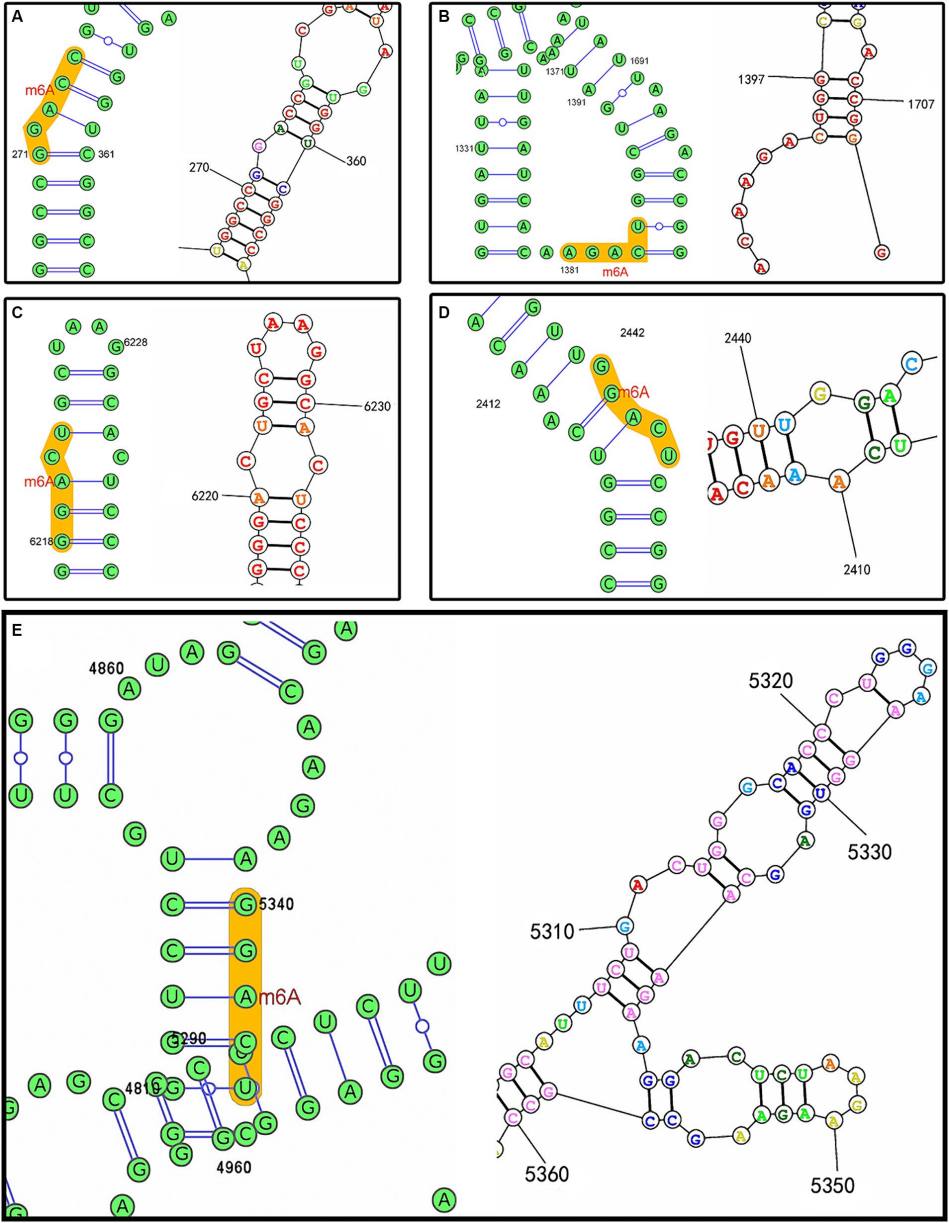
RNA secondary structures predicted by the SRAMP (left) and RNAstructure web-servers (right) based on the ALV-J prototype strain of HPRS-103 (GenBank accession number: Z46390.1). **(A)** Predicted m^6^A motif located at position 273. **(B)** Predicted m^6^A motif located at position 1,383. **(C)** Predicted m^6^A motif located at position 6,220. **(D)** Predicted m^6^A motif located at position 2,444. **(E)** Predicted m^6^A motif located at position 5,342.

The prediction results afforded by the SRAMP web-server indicated that the RNA secondary structure was mainly of two types, i.e.*, an “*A–U pair*” and a “*hairpin loop,*”* based on the core adenosine in the m^6^A motif. Among the 37 conserved motifs, 21 were of the A–U pair type, with the remaining motifs of the hairpin loop type, which included the loop adjacent to the G-C pair. The RNA secondary structures for each motif predicted by the SRAMP and RNAstructure web-servers were mostly consistent, such as the prediction of an A–U pair type for the motifs located at positions 273 (5′-LTR [U5]), 2,444 (coding region of gag [p 15]), and 6,220 (coding region of env [gp85]), although a hairpin loop type was predicted for the motif located at position 1,383 (coding region of gag [p27]). Moreover, the RNA secondary structures of the motif located at position 5,342 (overlapping coding regions of pol and env) were predicted to be of the A–U pair and hairpin loop types, respectively. The complex RNA secondary structures also indicated whether the structural change was related to the m^6^A modification (Figure 3).

## 4 Discussion

In recent years, the emergence of sequencing methods such as methylated RNA immunoprecipitation (MeRIP) and cross-linking and immunoprecipitation (CLIP) sequencing has given rise to a new layer of gene-expression regulation termed “epitranscriptomics,” which primarily focuses on the chemical alterations occurring on RNA molecules (28). However, these techniques can detect massive sequence segments containing m^6^A from the transcriptome, but cannot precisely identify the specific adenosine that undergoes methylation. Therefore, the use of computational tools to predict m^6^A sites from sequences would facilitate the rapid study of the m^6^a modification. From this viewpoint, the m^6^A motifs harbored in the genome of ALV-J strains available from GenBank were predicted using the SRAMP web-server. Consequently, more than 40 potential DRACH motifs were predicted in the genomes of these strains, among which 34.53 and 4.87% of the motifs had a “high confidence” and “very high confidence” grading, respectively. Studies related to the avian m^6^A modification revealed that “GGACU” was consistently regarded as the best and most highly enriched motif (29, 30). Moreover, “GGACU” was also preferred in the study of the transcriptome-wide dynamics of m^6^A methylation in chicken liver malignancies caused by ALV-J infection (22). Even though m6A motifs have not been determined for other subtypes of ALV, the consensus m6A sites across them would assist in unveiling the critical aspects of m6A modification in viral infection. According to the diversity analysis, the most common m^6^A-motif sequence of the DRACH motif of the ALV-J genome was also “GGACU,” which suggests the accuracy of this prediction, to some extent. Subsequently, although the 37 conserved motifs were predicted to be distributed in the U5 region of LTRs and all the major coding regions, they were enriched in the coding regions of the *p27*, *p68*, *p32*, and *gp85* genes. This high conservation further confirmed the prediction accuracy for the m^6^A motif.

In the ALV-J genome, the *gag* gene is relatively conserved and mainly encodes glycosylated proteins, including p19, p10, p27, p12, and p15 (7). p19, which is alternatively referred to as the viral matrix protein (MA), plays a crucial role in the viral outgrowth process; p12, also known as the nucleocapsid protein (NC), is involved in the processing and packaging of RNA; p15 is a protease (PR) that is involved in the cleavage of the precursors of viral genome-encoded proteins; and p27 is a capsid protein (CA) that serves as a common group-specific antigen of ALV, which distinguishes ALV from other oncogenic viruses because of its high conservation (4, 31, 32). Fourteen potential m^6^A motifs were predicted in the coding region of *p10*, *p12*, and *p15*, and eight were predicted for *p27*. Although p27 is relatively conserved between the different groups of ALV, whether the m^6^A modification occurred in all ALVs via these motifs warrants further analysis.

The *pol* gene exhibits conservation across the different ALV subgroups and encodes two essential enzymes, i.e., the reverse transcriptase (p68) and the integrase (p32): p68 can use viral RNA as a template for reverse transcription, to produce proviral DNA, which is then inserted into the host chromosome under the integrative action of p32 (33, 34). Moreover, 14 potential m^6^A motifs were predicted, half each for p68 and p32, including the motif locate at 3740 with a conservation percentage of 95.30% and a rating of “very high confidence.” The motifs enriched in p68 and p32 also indicated that the m^6^A modification may play a vital role in the reverse transcriptase integrase process during the infection course of ALV-J.

The *env* gene is highly variable across subtypes and encodes glycosylated vesicle membrane proteins, including the outer membrane protein gp85 and transmembrane protein gp37, linked by hydrogen and disulfide bonds; thus, *env* is a key gene in ALV tumorigenesis (35). In the current study, the number of conserved motifs of the *env* gene was relatively low possibly because of the mutability of the gene. However, motifs located in the coding region of *env* were also predicted in different ALV-J strains. It remains unclear whether these non-conservedly predicted motifs are useful and correlated with differences in strain pathogenicity. gp37 protein has two structurally important hydrophobic regions for fusion with cell membranes. Therefore, it has a significant impact on the fusion of viral vesicle membranes with cell membranes; gp85 contains a viral receptor determinant that recognizes a specific receptor on the membrane of target cells for infection. As the major antigen of viruses, gp85 not only determines the subgroup identity and host range of ALV but also induces specific neutralizing antibodies in chickens (36). Furthermore, another motif located at position 3,740 with a rating of “very high confidence” was detected in the overlapped coding region of *pol* and *env*. The RNA secondary structures of this motif were predicted to be A–U pair or hairpin loop, which also indicated the complex RNA secondary structures induced by the sequences flanking each m^6^A motif. As reported previously, m^6^A stacking at the end of a hairpin loop stabilizes the loop, whereas the *N*^6^-methylation of an A–U pair in the middle of a helix would reduce the stability of helix folding (27). Refer to the conserved motifs predicted in ALV-J genomes, they mainly contained A–U pair or hairpin loop, and m^6^A modification of the unpaired adenosines attached in each helix have been confirmed to increase folding stability (27). Therefore, secondary structure prediction may help determine the structure–function relationships, thus promoting the understanding of the roles of m^6^A modifications and improving their prediction accuracy.

In this study, m^6^A modification motifs were predicted in different ALV-J strains, and their conservation was analyzed. Moreover, the RNA secondary structure of some representative motifs was determined. Although these motifs warrant further identification, a systematic comparative analysis would facilitate the rapid investigation of m^6^a modifications in ALV-J.

## Data availability statement

The datasets presented in this study can be found in online repositories. The names of the repository/repositories and accession number(s) can be found in the article/Supplementary material.

## Author contributions

JJ: Investigation, Writing – original draft. XM: Data curation, Writing – original draft. SX: Software, Writing – original draft. XX: Data curation, Methodology, Writing – original draft. ZZ: Methodology, Visualization, Writing – original draft. LY: Supervision, Writing – review & editing. QX: Writing – review & editing. YB: Writing – review & editing.

## Footnotes

^1^ http://www.cuilab.cn/sramp/

^2^ https://www.chiplot.online/lollipop_plot.html

^3^ https://rna.urmc.rochester.edu/RNAstructureWeb/index.html
