# Supplementary material for: Conservation and distribution of the DRACH motif for potential m6A sites in avian leukosis virus subgroup J
Source: Front Vet Sci. 2024 Apr 12;11:1374430. doi: 10.3389/fvets.2024.1374430 (PMC11046932; doi:10.3389/fvets.2024.1374430)
Supplement: Supplementary file 1 [file Table_1.DOCX]

Supplementary Material

Conservation and distribution of the DRACH motif for potential m6A sites in avian leukosis virus subgroup J

Jun Ji^1,^* , Xinhao Mu^1^, Shuqi Xu^1^, Xin Xu^1^, Zhibin Zhang^1^, Lunguang Yao^1^, Qingmei Xie^2^, Yingzuo Bi^2^

**Correspondence:** Corresponding Author: jijun020@126.com

# Supplementary Table 1. Clinical details and m6A predictions of ALV-J strains obtained from the GenBank

| **Accession no.** | **Strains** | **Time** | **Countries** | **Rating scores** | | | | **Total** |
| --- | --- | --- | --- | --- | --- | --- | --- | --- |
|  |  |  |  | **Low** | **Moderate** | **High** | **Very High** |  |
| Z46390 | HPRS103 | 1988 | UK | 11 | 25 | 18 | 2 | 56 |
| DQ115805 | NX0101 | 2001 | China | 12 | 18 | 16 | 2 | 48 |
| KP284572 | PDRC-59831 | 2007 | USA | 12 | 24 | 21 | 3 | 60 |
| MN956379 | PK19FA01 | 2018 | Pakistan | 8 | 24 | 24 | 2 | 58 |
| KU937324 | GX14YYD2 | 2014 | China | 14 | 19 | 20 | 4 | 57 |
| KX037423 | GX14ZS14 | 2014 | China | 14 | 16 | 21 | 3 | 54 |
| MF461280 | GX14YYA1 | 2014 | China | 12 | 21 | 15 | 3 | 51 |
| MT108923 | GX191201 | 2019 | China | 14 | 20 | 20 | 2 | 56 |
| MT119963 | GX-RJ01 | 2020 | China | 9 | 18 | 22 | 2 | 51 |
| MT512432 | GX-2020-01 | 2020 | China | 11 | 18 | 21 | 2 | 52 |
| KY767730 | SDAUAJ-3 | 2014 | China | 12 | 20 | 20 | 2 | 54 |
| HM235665 | HAY013 | 2008 | China | 15 | 16 | 22 | 2 | 55 |
| HM235667 | JS-nt | 2003 | China | 15 | 14 | 22 | 3 | 54 |
| MN735308 | JS14NT01 | 2014 | China | 13 | 19 | 18 | 2 | 52 |
| KF562373 | BR119 | 2011 | China | 15 | 14 | 24 | 2 | 55 |
| HM776937 | SVR807 | 2008 | Russia | 11 | 19 | 23 | 2 | 55 |
| MN735293 | GX14NN02 | 2014 | China | 13 | 19 | 21 | 3 | 56 |
| KR025484 | GX14YL03 | 2014 | China | 15 | 22 | 14 | 4 | 55 |
| MN735294 | GX15JL01 | 2015 | China | 14 | 23 | 17 | 2 | 56 |
| JF932002 | CAUTS01 | 2009 | China | 9 | 18 | 21 | 4 | 52 |
| KM376510 | HLJ13SH01 | 2013 | China | 15 | 19 | 20 | 3 | 57 |
| GU982308 | JS09GY3 | 2009 | China | 14 | 23 | 11 | 4 | 52 |
| GU982310 | JS09GY6 | 2009 | China | 13 | 23 | 14 | 3 | 53 |
| JN624878 | JL093-1 | 2009 | China | 13 | 19 | 18 | 4 | 54 |
| JF932001 | CAUSY01 | 2009 | China | 10 | 16 | 16 | 4 | 46 |
| JX848322 | WB11098J | 2011 | China | 15 | 14 | 21 | 4 | 54 |
| KT156668 | SDAU1005 | 2010 | China | 18 | 17 | 18 | 3 | 56 |
| KU159178 | SDAU1102 | 2010 | China | 16 | 18 | 19 | 3 | 56 |
| JN389517 | sdau1001 | 2010 | China | 12 | 20 | 18 | 3 | 53 |
| JN624879 | SD09DP03 | 2009 | China | 12 | 19 | 17 | 2 | 50 |
| JN389518 | sdau1002 | 2010 | China | 11 | 20 | 18 | 3 | 52 |
| KY980662 | SDAU1706 | 2017 | China | 10 | 18 | 24 | 4 | 56 |
| KY980657 | SDAU1701 | 2017 | China | 10 | 20 | 22 | 4 | 56 |
| KY980661 | SDAU1705 | 2017 | China | 13 | 19 | 22 | 3 | 57 |
| KY980659 | SDAU1703 | 2017 | China | 12 | 19 | 27 | 3 | 61 |
| KF562374 | SD110503 | 2011 | China | 13 | 25 | 18 | 2 | 58 |
| JX254901 | GD1109 | 2011 | China | 11 | 19 | 19 | 4 | 53 |
| JF932003 | CAUXT01 | 2009 | China | 10 | 19 | 20 | 3 | 52 |
| JF932004 | CAUYL01 | 2009 | China | 12 | 20 | 16 | 3 | 51 |
| JF932000 | CAUHM01 | 2009 | China | 6 | 20 | 15 | 4 | 45 |
| MN956380 | PK19SA01 | 2018 | Pakistan | 10 | 19 | 22 | 2 | 53 |
| JF951728 | MRL905 | 2009 | Russia | 17 | 22 | 20 | 3 | 62 |
| JQ935966 | CLB908U | 2009 | Russia | 12 | 20 | 19 | 2 | 53 |
| JX855935 | CLB908M | 2009 | Russia | 10 | 19 | 25 | 2 | 56 |
| KC711043 | WN100401 | 2010 | China | 12 | 21 | 21 | 4 | 58 |
| JX423792 | GDQY1201 | 2012 | China | 16 | 23 | 16 | 3 | 58 |
| JX453210 | GDKP1202 | 2012 | China | 16 | 23 | 16 | 3 | 58 |
| KC149971 | SCAU11-XG | 2011 | China | 13 | 17 | 17 | 2 | 49 |
| KC149972 | SCAU11-H | 2011 | China | 16 | 21 | 12 | 1 | 50 |
| KU156826 | GDQJ2 | 2014 | China | 11 | 18 | 20 | 2 | 51 |
| KU170199 | GDQJ-4 | 2014 | China | 13 | 20 | 21 | 2 | 56 |
| KU170200 | GDQJ-5 | 2014 | China | 8 | 22 | 16 | 2 | 48 |
| KU500030 | GD13GZ | 2013 | China | 13 | 20 | 19 | 3 | 55 |
| KU500031 | GD13HY | 2013 | China | 15 | 21 | 19 | 2 | 57 |
| KU500032 | GD14J2 | 2014 | China | 11 | 22 | 17 | 3 | 53 |
| KU500033 | GD1406-H | 2014 | China | 14 | 19 | 19 | 3 | 55 |
| KU500034 | GD1407 | 2014 | China | 17 | 18 | 15 | 2 | 52 |
| KU500035 | GD1407-L | 2014 | China | 12 | 20 | 21 | 2 | 55 |
| KU500036 | GD1408-1 | 2014 | China | 8 | 23 | 18 | 3 | 52 |
| KU500037 | GD1408-2 | 2014 | China | 14 | 19 | 15 | 2 | 50 |
| KU500038 | GD1411-1 | 2014 | China | 9 | 16 | 15 | 3 | 43 |
| KU500039 | GD1411-2 | 2014 | China | 10 | 19 | 18 | 3 | 50 |
| KU500040 | GD1411-3 | 2014 | China | 12 | 23 | 18 | 3 | 56 |
| KU500041 | GD1411-4 | 2014 | China | 9 | 23 | 19 | 3 | 54 |
| KX034517 | GX14LT07 | 2014 | China | 15 | 23 | 20 | 3 | 61 |
| KX611834 | M180 | 2016 | China | 13 | 21 | 24 | 4 | 62 |
| MK683478 | GDHN-YM1 | 2018 | China | 15 | 16 | 21 | 2 | 54 |
| MK683479 | GDHN-YM2 | 2018 | China | 17 | 21 | 20 | 2 | 60 |
| MK683480 | GDHN-YH1 | 2018 | China | 18 | 17 | 12 | 2 | 49 |
| MK683481 | GDHN-YH2 | 2018 | China | 15 | 20 | 23 | 2 | 60 |
| MK940585 | GDYH-B1 | 2018 | China | 12 | 22 | 14 | 2 | 50 |
| MN893850 | GD19GZ01 | 2019 | China | 19 | 16 | 21 | 2 | 58 |
| MN893851 | GD19GZ02 | 2019 | China | 18 | 18 | 20 | 2 | 58 |
| MT175600 | SCAU1903 | 2019 | China | 11 | 19 | 21 | 3 | 54 |
| MT538237 | GD16FS01 | 2016 | China | 18 | 25 | 19 | 2 | 64 |
| MT538238 | GD17HZ01 | 2017 | China | 16 | 20 | 14 | 3 | 53 |
| MT538239 | GD18FS01 | 2018 | China | 18 | 20 | 18 | 2 | 58 |
| MT538240 | GD18HZ01 | 2018 | China | 8 | 25 | 16 | 2 | 51 |
| MT538241 | GD18JM01 | 2018 | China | 14 | 24 | 17 | 2 | 57 |
| MT538242 | GD18SG01 | 2018 | China | 10 | 18 | 23 | 2 | 53 |
| MT538243 | GD18ZH01 | 2018 | China | 14 | 24 | 16 | 2 | 56 |
| MT538244 | GD18ZJ01 | 2018 | China | 7 | 28 | 14 | 2 | 51 |
| MT538245 | GD19FS01 | 2019 | China | 14 | 22 | 16 | 4 | 56 |
| MT538247 | GD19HZ02 | 2019 | China | 11 | 21 | 23 | 3 | 58 |
| MT538248 | GD19ZH01 | 2019 | China | 15 | 18 | 17 | 2 | 52 |
| MT538251 | GD16JM01 | 2016 | China | 18 | 23 | 18 | 2 | 61 |
| MT538253 | GD17FS01 | 2017 | China | 11 | 21 | 23 | 2 | 57 |
| KU934276 | GX15MM6-2 | 2015 | China | 15 | 20 | 16 | 2 | 53 |
| KU997685 | GX14HG01 | 2014 | China | 12 | 18 | 20 | 2 | 52 |
| KX058878 | GX14HG04 | 2014 | China | 10 | 22 | 19 | 2 | 53 |
| MN066140 | GX18NN02 | 2018 | China | 14 | 17 | 21 | 2 | 54 |
| MN066141 | GX18NN01 | 2018 | China | 10 | 19 | 21 | 2 | 52 |
| MN066142 | GX17YL05 | 2017 | China | 13 | 17 | 19 | 2 | 51 |
| MN066143 | GX17YL01 | 2017 | China | 14 | 20 | 17 | 2 | 53 |
| MN066144 | GX17NN06 | 2017 | China | 12 | 19 | 17 | 3 | 51 |
| MN066145 | GX17NN05 | 2017 | China | 12 | 18 | 20 | 3 | 53 |
| MN066146 | GX16ZS01 | 2016 | China | 11 | 22 | 18 | 3 | 54 |
| MN066147 | GX16YL02 | 2016 | China | 15 | 18 | 19 | 3 | 55 |
| MN066148 | GX16YL01 | 2016 | China | 14 | 19 | 18 | 3 | 54 |
| MN066149 | GX16MM92 | 2016 | China | 11 | 18 | 21 | 2 | 52 |
| MN066150 | GX15MM61 | 2015 | China | 17 | 15 | 22 | 3 | 57 |
| MN066151 | GD15MM02 | 2015 | China | 15 | 19 | 13 | 3 | 50 |
| MN066152 | GD15MM01 | 2015 | China | 19 | 12 | 21 | 4 | 56 |
| MN066153 | GX14NN02 | 2014 | China | 14 | 18 | 16 | 2 | 50 |
| MN066154 | GX14NN01 | 2014 | China | 15 | 22 | 18 | 3 | 58 |
| MN735292 | GX14NN01 | 2014 | China | 11 | 25 | 15 | 3 | 54 |
| MN735295 | GX16NN03 | 2016 | China | 17 | 19 | 16 | 3 | 55 |
| MN735296 | GX16NN04 | 2016 | China | 11 | 24 | 20 | 2 | 57 |
| MN735297 | GX16YL01 | 2016 | China | 12 | 23 | 16 | 3 | 54 |
| MN735309 | GX16NN05 | 2016 | China | 12 | 22 | 17 | 3 | 54 |
| JN624880 | HLJ09MDJ-1 | 2009 | China | 8 | 16 | 22 | 4 | 50 |
| HM235668 | NHH | 2007 | China | 16 | 19 | 21 | 3 | 59 |
| HQ900844 | SCAU-HN06 | 2006 | China | 16 | 19 | 19 | 4 | 58 |
| MN735299 | HN17ZZ01 | 2017 | China | 15 | 23 | 22 | 1 | 61 |
| MN735300 | HN17ZZ02 | 2017 | China | 15 | 26 | 20 | 2 | 63 |
| HM235670 | YZ9902 | 1999 | China | 7 | 19 | 19 | 2 | 47 |
| MN735301 | JS13NT01 | 2013 | China | 20 | 21 | 20 | 2 | 63 |
| MN735302 | JS14NT02 | 2014 | China | 19 | 20 | 14 | 3 | 56 |
| MN735303 | JS18YZ01 | 2018 | China | 9 | 21 | 16 | 3 | 49 |
| MN735304 | JS18YZ02 | 2018 | China | 16 | 20 | 18 | 2 | 56 |
| MN735305 | JS18YZ03 | 2018 | China | 11 | 23 | 15 | 3 | 52 |
| FJ216405 | SD07LK1 | 2007 | China | 17 | 24 | 16 | 2 | 59 |
| KY980658 | SDAU1702 | 2017 | China | 10 | 21 | 21 | 2 | 54 |
| KY980660 | SDAU1704 | 2017 | China | 9 | 20 | 24 | 2 | 55 |
| HQ425636 | SCDY1 | 2009 | China | 11 | 20 | 18 | 3 | 52 |
| JQ396302 | SCGS-1 | 2010 | China | 15 | 20 | 18 | 2 | 55 |
| AY027920 | ADOL-7501 | 1997 | USA | 7 | 23 | 21 | 2 | 53 |
| MT538250 | GD16HZ01 | 2016 | China | 12 | 16 | 23 | 3 | 54 |
| KU254611 | GDQJ-1 | 2014 | China | 15 | 21 | 21 | 2 | 59 |
| MT538249 | GD19ZH02 | 2019 | China | 12 | 17 | 14 | 3 | 46 |
| MT538252 | GD16HZ02 | 2016 | China | 10 | 19 | 24 | 3 | 56 |
| MT538246 | GD19HZ01 | 2019 | China | 15 | 18 | 20 | 3 | 56 |
| MN735298 | HB18XH01 | 2018 | China | 15 | 19 | 18 | 3 | 55 |
| MN735307 | SH18JY02 | 2018 | China | 16 | 16 | 17 | 3 | 52 |
| MN735306 | SH18JY01 | 2018 | China | 12 | 17 | 17 | 3 | 49 |
| OP918846 | LY2021J | 2021 | China | 14 | 18 | 22 | 3 | 57 |
| OP508143 | RKZ-1 | 2022 | China | 14 | 16 | 14 | 2 | 46 |
| OP856678 | RKZ-2 | 2022 | China | 10 | 19 | 13 | 2 | 44 |
| OP382470 | HB2021017 | 2021 | China | 14 | 22 | 15 | 2 | 53 |
| ON840093 | HB2020 | 2020 | China | 8 | 18 | 21 | 2 | 49 |
| OK507207 | DPRJ21 | 2020 | India | 11 | 24 | 17 | 2 | 54 |
| OL799231 | JSYC2106-1 | 2021 | China | 16 | 18 | 18 | 4 | 56 |
| OL799232 | JSYC2106-2 | 2021 | China | 15 | 19 | 18 | 4 | 56 |
| MW891539 | CAU2020 | 2020 | China | 12 | 19 | 18 | 3 | 52 |
| MW891540 | CAU2019 | 2019 | China | 9 | 24 | 22 | 2 | 57 |
| MT863859 | GD19GZ03 | 2019 | China | 15 | 22 | 17 | 1 | 55 |
| MT863860 | GD19GZ04 | 2019 | China | 15 | 19 | 24 | 4 | 62 |
| MZ856317 | GD1804J6 | 2018 | China | 9 | 22 | 18 | 3 | 52 |
| MK944404 | LH20180301 | 2019 | China | 10 | 24 | 17 | 2 | 53 |
